# Effect of glass-ionomer cement as an intra-canal barrier in post space prepared teeth: An *in vitro* study

**DOI:** 10.4103/0972-0707.55620

**Published:** 2009

**Authors:** Rajakumar Vijay, R Indira

**Affiliations:** Department of Conservative Dentistry and Endodontics, Ragas Dental College and Hospital, Chennai, India

**Keywords:** Coronal microleakage;, glass ionomer, intra canal barrier, post space

## Abstract

**Aim:**

To evaluate the bacterial microleakage across remaining Gutta-percha in teeth prepared for post space with and without the use of an intracanal glass ionomer barrier.

**Materials and Methods:**

Forty freshly extracted intact human mandibular premolars with single canal were instrumented, obturated with Gutta-percha and AH plus sealer and post spaces were created. Teeth were assigned into experimental groups as follows: Group I – 3 mm of Gutta-percha, Group II – 4 mm of Gutta-percha, Group III – 3 mm of Gutta-percha with 1 mm of Vitrebond as barrier, Group IV – 4 mm of Gutta-percha with 1mm of Vitrebond as barrier. The roots were suspended in Rogosa SL broth and 50 μl of lyophilized Lactobacilli Casei was inoculated as the microbial marker. The mean days taken for the broth to turn turbid were tabulated. The values were statistically analyzed using one way ANOVA and Tukey's HSD test.

**Results:**

At the end of 64 days, the mean and standard deviation of the number of days for the broth to turn turbid was: Group I – 20.50, (SD - 3.96). Group II – 25.43, (SD - 4.83), Group III – 38.63, (SD - 9.36), and Group IV – 53.50, (SD - 11.15)

**Conclusion:**

Vitrebond could be used as an intracanal barrier to provide a superior coronal seal in teeth requiring post and core.

## INTRODUCTION

Coronal leakage of the root canal filling is considered to be an important cause of failure of root canal therapy. Torabinejad *et al.* showed that more than 50% root canals were completely contaminated when the coronal surfaces of their fillings were exposed to Staphylococcus epidermidis. Microleakage due to missing or compromised coronal seal is often implicated in the failure of root canal therapy.[1]

Swanson and Madison reported that the exposure of the coronal segments of obturated root canals to artificial saliva resulted in recontamination of 79% to 85% of the root canal system in as little as 3 days.[2] They found that all root canals were contaminated in less than 30 days. Investigators began to study the barriers that could help prevent coronal microleakage. Pisano *et al.* showed that an intra-orifice barrier provided a secondary seal for obturated teeth without restoration.[3] Gutta-percha remaining after post space preparation does not provide seal equivalent to the intact root canal filling. Chailertvanitkul *et al.* demonstrated that a glass ionomer cement (Vitrebond) intra-orifice barrier provided an adequate barrier for endodontically treated teeth.[4] The aim of this study is to demonstrate the bacterial microleakage of the remaining Gutta-percha in post space prepared teeth with and without the use of an intra-canal glass ionomer cement as a barrier (Vitrebond).

## MATERIALS AND METHODS

Forty freshly extracted human mandibular intact premolar teeth with single canal were stored with 0.2% sodium azide for 4 weeks. Teeth were examined for fractures or defects under transillumination by 3× magnification loupe and were eliminated from the study. The apical terminus of each tooth was determined by passing #15 file through apical foramen until visible and working length was set short of that point. Canals were prepared using crown down technique; 5% NaOCl and EDTA was used alternatively through out the instrumentation to remove the smear layer. Canals were dried using sterile paper points and obturated with 2% Gutta-percha by cold lateral compaction using AH Plus as sealer material. Post space was created using warm pluggers and Pesso Reamer no. 2 and no. 3, and the excess sealer was removed. A minimum of 3 mm thickness of residual Gutta-percha, which was confirmed using radiovisiography (RVG), was maintained in all the samples of different groups.

All the teeth were decoronated at 1 mm coronal to the cemento enamel junction (CEJ) and were grouped as follows: Group I – 3 mm of Gutta-percha, Group II – 4 mm of Gutta-percha, Group III – 3 mm of Gutta-percha plus 1 mm of Vitrebond as barrier, and Group IV – 4 mm of Gutta-percha plus 1 mm of Vitrebond as barrier. Vitrebond (3MESPE) was mixed according to manufacturers recommendations and directly placed over the remaining Gutta-percha using Centrix syringe. Each specimen was radiographed to verify the thickness of Gutta-percha and Vitrebond. Vitrebond was light cured for 60 s as recommended, and the specimens were placed in 100% humidity for 48 h for allowing the sealer to set. To ensure curing of cement, a light transmitting post was placed to pass the light within the canal space. Since the material is photosensitive, when irradiated by a curing unit, photoinitiator cross-linking of the polymer chain occurs through free radical methacrylate polymerization. Thus, light curing is considerably faster than the acid base reaction, which results in the command setting of the mixed Vitrebond material. Two positive controls – Instrumented, but nonobturated. Two negative controls – Post space prepared with 4 mm of remaining Gutta-percha, and the root was completely sealed with cyanoacrylate and nail polish.

A presterilized Scintillation vial with a plastic top was used, and a hole was made on the center of the plastic top so that one quarter inch of a vinyl tube would snugly fit through the hole and the junctions were sealed with cyanoacrylate. Each root was coronally reduced to produce convergent axial walls, which produced better adaptation to the vinyl tubing as well as increased surface area for bonding and sealing the tooth/tubing interface. Two inch sections of the vinyl tubing were bonded over the coronal aspect of the root with cyanoacrylate except for the apical 3 mm. Experimental teeth and controls were coated with cyanoacrylate and then two coats of nail polish to block the lateral and accessory canals. The specimens were suspended in the vials with sterile Rogosa SL broth that covered the root ends. The top end of the vinyl tubing was plugged with cotton and removed during inoculation. The entire set up was kept in the laminar flow under ultraviolet light over night to ensure sterilization before inoculation.

Lyophilized Lactobacillus casei (ATCC # 11578) was suspended into the prepared media and incubated over night at 37°C. The culture suspension was adjusted to match the turbidity equivalent to 0.5 McFarland standards (1.5 × 10^8^ CFU ml^−1^). 50 μl of the inoculum was transferred to presterilized individual scintillation vial through the vinyl tubing that led into root canal and was changed every 5 days. Samples were incubated at 37°C. All the microbiological procedures were conducted under a laminar hood. If the broth turned turbid, the microorganism had penetrated the entire length of the root canal. The day number that the sample turned turbid were recorded, and the experiment was terminated on day 64 when the broth of the last experimental tooth turned turbid. The presence of Lactobacilli in each sample was verified by Gram stain.

## RESULTS

At the end of 64 days, the mean and standard deviation of the number of days for the broth to turn turbid was: Group I – 20.50, (SD - 3.96). Group II – 25.43, (SD – 4.83), Group III – 38.63, (SD - 9.36), and Group IV – 53.50, (SD - 11.15) [Table 1]. In the positive control group, all the specimens turned turbid, whereas in the negative control group, none of the specimens turned turbid throughout the experiment period of 64 days. The results of the present study were subjected to statistical analysis to interpret the significant differences between various specimens in each group and also between the groups. One-way ANOVA, followed by Tukey's HSD post-hoc tests were used for statistical analysis in the present study [Table 1]. The results of the present investigation revealed that there was no statistical difference between Group I (3 mm of Gutta-percha) and Group II (4 mm of Gutta-percha), However, there was statistically significant difference between the Group II (4 mm of Gutta-percha) and Group III (3 mm of Gutta-percha plus 1 mm of Vitrebond) and Group IV (4mm of Gutta-percha plus 1 mm.

**Table 1:** Mean number of days for the broth to turn turbid

Groups	No. of days		
	
	Mean	SD	P value
Group I	20.50^a^	3.96	< 0.001**
Group II	25.43^a^	4.83	
Group III	38.63^b^	9.36	
Group IV	53.50^c^	11.15	

** Denotes significance at 1% level.

a Denote significance at 5% level.

b Denote significance at 5% level.

c Denote significance at 5% level.

## DISCUSSION

Bacteria has shown to play an important role in endodontic failures. There exist a great association between the practice of endodontics and the science of microbiology. The vast majority of diseases of dental pulp and the periradicular tissues are associated with micro-organisms. Maintaining a coronal seal and placement of a definitive restoration should be considered as an essential component of successful endodontic treatment. Wolcott, Hicks and Himel, in their study, concluded that glass ionomer is an effective intra-orifice barrier to prevent microbial leakage, whereas teeth with only Gutta-percha and sealer showed extensive leakage throughout their experimental period. Wolcott, Hicks and Himel specify the following criteria for an intra-orifice barrier: it should be easily placed by the operator, it should bond to the tooth structure (retentiveness), it should seal effectively against coronal microleakage, it should be easily distinguished from the natural tooth structure, and it should not interfere with the final restoration of access preparation.[5]

Chailertvanitkul *et al.* suggested that microorganisms might be more appropriate than dyes or isotopes for studying leakage. *Hence, in the present study, a bacterial leakage model was selected to prove the appearance of turbidity, which is in accordance to the study by Pisano et al.* The experimental leakage apparatus (Scintillation vial) used in this study was a modification of the model designed by Pisano *et al*.[6]

Lactobacillus casei was used as test organism as it is a Gram positive facultative anaerobic bacterium that can be easily cultured and it is most commonly found in contaminated root canals. Since it can be selectively cultured on agar plates and in Rogosa broth, it eliminated the risk of cross contamination from other microbes because of its low pH. Therefore, if turbidity appears, it is because of the lactobacillus penetration of the root canal system and not from other sources. Bacterial studies have been qualitative rather quantitative. If only one bacterium passes through the obturated root canal, it may multiply in the enriched broth and cause turbidity. The present study was based on all or none qualitative approach, i.e., leakage was registered positive only when turbidity in the vial indicated complete penetration of the residual Gutta-percha in the canal system.

Celik *et al.* showed that the coronal leakage of the root canal filling is considered as an important cause for failure of root canal therapy. A coronal restoration that fails to provide a seal could permit the movement of microorganisms or their toxins along the canal walls or through voids in the root canal filling to the periapical tissues.[7] Wu *et al.* studied microleakage along apical root fillings and cemented posts, and they found that apical 4 mm of root canal filling remaining after post space preparation leaks statistically and more significantly than the original full length root canal filling. The leakage created by the removal of the coronal part of root canal filling during post space preparation may be compensated by the cemented posts.[8]

James *et al.* evaluated the effectiveness of three pigmented glass ionomer cements used as intracanal barriers to prevent coronal microleakage. From the results of their study, the teeth without an intra-orifice barrier were found to leak significantly more than teeth with Vitrebond (intra-orifice barrier) and also it indicates that a glass ionomer is an effective barrier to prevent microbial leakage, whereas teeth with only Gutta-percha and sealer showed extensive leakage after the 90 day experimental period.[9] Even in the present study, specimens of Group I and Group II leaked significantly more than Group III and Group IV specimens with a barrier, thereby suggesting the importance of intracanal barrier. In the present study, Group IV specimens showed the least leakage, thereby suggesting that the residual length of Gutta-percha must be at least 4 mm.

Endodontically treated teeth are often restored by a post and a core. The main purpose of the post is to retain the permanent restoration and disperse the forces along the root. Nevertheless the seal provided by a full length root canal filling may be compromised by the post space preparation. Therefore, Moshonov *et al.* concluded that the remaining Gutta-percha should be in contact with the post.[10] The time between obturation and placement of the permanent restoration is critical to prevent the recontamination of the remaining apical Gutta-percha. Hence, particularly post spaces should be restored immediately because of the difficulties associated with maintaining the temporary seal to prevent recontamination of the residual Gutta-percha.[11]

The results of the present study revealed that Group IV specimens (i.e., those with 4 mm of Gutta-percha plus 1 mm of Vitrebond) showed the least leakage, followed by Group III (3 mm of Gutta-percha plus 1 mm of Vitrebond).Group I and Group II leaked earlier than Group III and Group IV. Group I (3 mm of Gutta-percha) leaked faster than Group II (4 mm of Gutta-percha).Thus, in this study, Vitrebond proved to be an acceptable intracanal barrier and should provide a superior coronal seal. In this study, teeth with 4 mm of Gutta-percha plus 1 mm of Vitrebond were nonturbid for a mean of 53.50 days, while teeth with 3 mm of Gutta-percha turned turbid at 20.50 days, and the difference was statistically significant (*P* < 0.05).

Thus, in the compromised clinical situation of a short tooth requiring a post and core, which allowed 4 mm of Gutta-percha, a glass ionomer barrier over the Gutta-percha could reduce the risk of contamination of the apical Gutta-percha by bacterial microleakage. The present study was limited to a single microorganism and was done *ex-vivo*. Further studies with different microorganisms present in the oral cavity and in vivo studies are required to extrapolate the results of the present study. In this study, dual cure/(resin modified) glass ionomer cement was used as a intra-coronal barrier. With the advent of newer materials, future research needs to be directed aiming towards the goal of achieving an impervious seal and successful post endodontic restoration.

## CONCLUSION

Thus, in this study, Vitrebond proved itself as an effective intracanal barrier that could provide a superior coronal seal. Further it can be inferred from this study that when the length of the residual Gutta-percha was 4 mm, it could minimize the rate of bacterial leakage better in comparison to the 3 mm of residual Gutta-percha. Lack of coronal seal can be a detrimental factor contaminating the obturated root canal, complicating the treatment outcome. From this perspective, it is important that the intracanal barrier should provide adequate coronal seal and prevent the entry of bacterial toxins into the root canal that compromises the success of the root canal treatment.
